# A Rare Cystic Fibrosis Transmembrane Conductance Regulator (CFTR) Mutation Associated With Typical Cystic Fibrosis in an Arab Child

**DOI:** 10.7759/cureus.13526

**Published:** 2021-02-24

**Authors:** Aji Mathew, Mohammed Dirawi, Ahmad Abou Tayoun, Rizwana Popatia

**Affiliations:** 1 Pediatric Pulmonology, Al Jalila Children's Hospital, Dubai, ARE; 2 Pediatrics, Al Jalila Children's Hospital, Dubai, ARE; 3 Genetics, Al Jalila Children's Hospital, Dubai, ARE; 4 Pediatric Medicine, Al Jalila Children's Hospital, Dubai, ARE

**Keywords:** cftr gene, pancreatic insufficiency

## Abstract

Cystic fibrosis (CF) is a progressive genetic disorder, inherited by the autosomal recessive mode of inheritance and more frequently seen in the Caucasian population with a carrier rate of 1:29 in Caucasian-Americans. Over 1800 cystic fibrosis transmembrane conductance regulator (CFTR) gene mutations have been identified so far and the delta F 508 del mutation is the most common mutation. Gene sequencing and deletion/duplication analysis can detect mutations in 99% of people with a clinical diagnosis of CF. However, diagnostic testing can be challenging, as screening tests may be inconclusive and the routine gene mutation panel analysis may be negative due to some rare or undocumented mutations.

We report a case of a two-year-old boy of Palestinian-Lebanese descent, with a history of raised immunoreactive trypsin test (IRT), positive sweat test, and phenotypical CF manifestations, found to have rare CF apparent homozygous *CFTR* (NM_000492.3) variant, c.3623del (p.Gly1208AlafsX3). In our case, genetic testing for 139 mutations done in Germany could not identify any defect. Only CFTR gene sequencing identified the above pathogenic variant. This reinforces the practice for a broad range of CFTR mutation analyses to detect ethnic-specific rare variants. This is the second case of this particular genetic mutation identified and the first to be reported in detail.

## Introduction

Cystic fibrosis (CF) is a progressive genetic disorder, inherited by the autosomal recessive mode of inheritance and more frequently seen in the Caucasian population with a carrier rate of 1:29 in Caucasian-Americans [1]. Over 1800 cystic fibrosis transmembrane conductance regulator (CFTR) gene mutations have been identified so far, and the delta F508 del mutation is the most common mutation [2]. In the Arab population, CF is less common and the exact incidence is not yet determined. The F508del mutation along with some other native Arab mutations are described to be common mutations [3-4]. CFTR gene mutations lead to a dysfunctional or absent CFTR protein, which, in turn, causes the inability of movement of chloride ions to the cell surface along with water, resulting in thick and sticky mucus in various organs [5].

Clinical manifestations vary from recurrent lung infections, malnutrition and poor growth, liver disease, and impaired reproduction and infertility. The diagnosis of cystic fibrosis usually encompasses a newborn screening test, a sweat test, genetic testing, and clinical evaluation. Gene sequencing and deletion/duplication analysis can detect mutations in 99% of people with a clinical diagnosis of CF. However, diagnostic testing can be challenging, as screening tests may be inconclusive and the routine gene mutation panel analysis may be negative due to some rare or undocumented mutations.

We report a case of a two-year-old boy of Palestinian-Lebanese descent, with a history of raised immunoreactive trypsin test (IRT), positive sweat test, and phenotypical CF manifestations, found to have a rare CF apparent homozygous CFTR (NM_000492.3) variant, c.3623del (p.Gly1208Alafsx3), with legacy name 3755 delG. This case was missed in a limited CF genetic mutation analysis and is so far the second case described in the literature.

## Case presentation

A two-year-old boy of Palestinian-Lebanese descent was initially seen in our clinic at five months of age. His perinatal history was unremarkable except for high IRT levels (156) in newborn screening. His parents are distant relatives, and his siblings were normal. Sweat chloride was 124 mmol/L, and he had very low stool pancreatic elastase.

**Figure 1:**
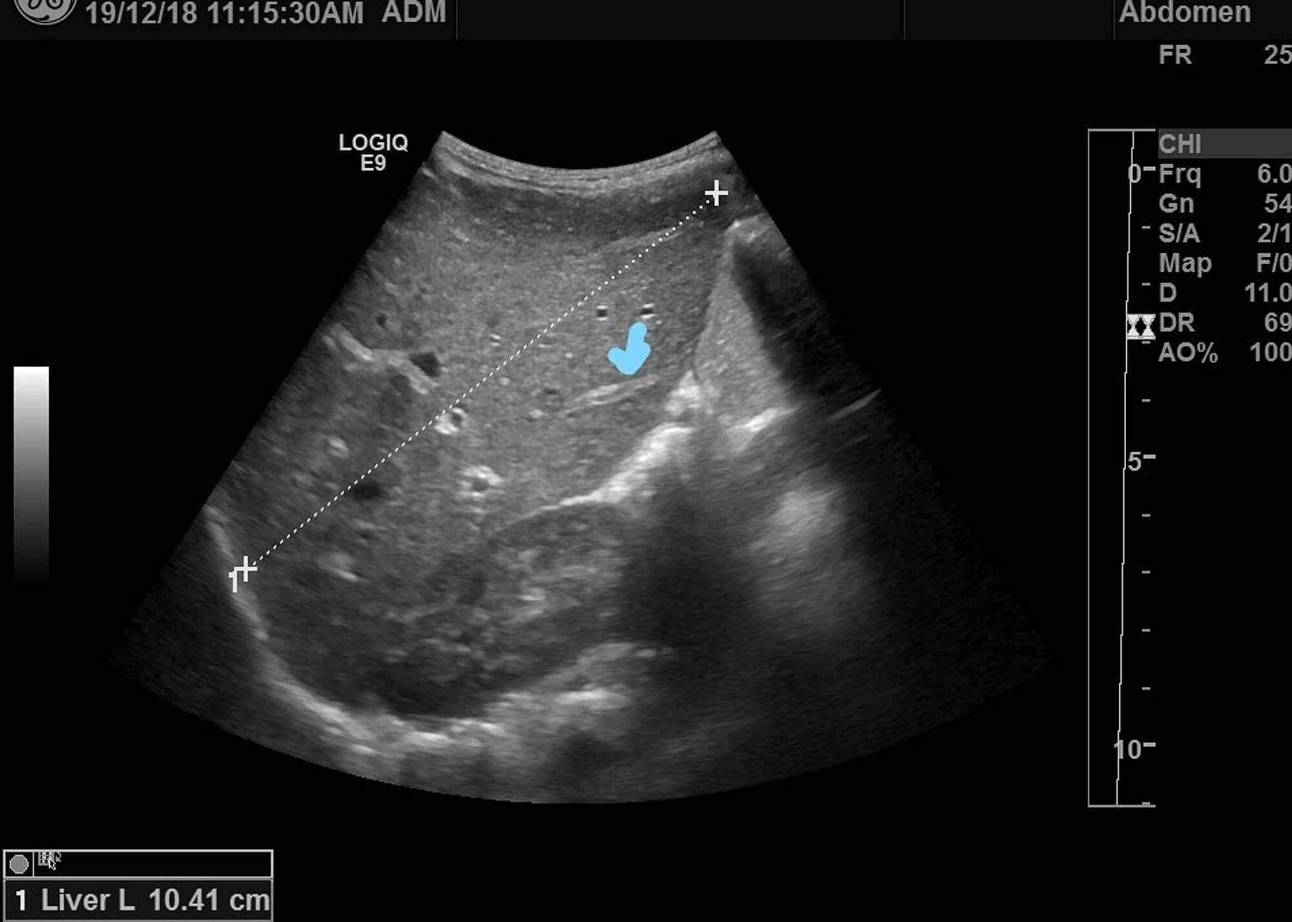
Mildly enlarged liver with prominent periportal tracts

Based on this, CFTR mutation for 139 mutations was initially done in Germany, and it did not identify any disease-causing mutations. Clinical examination was unremarkable except for failure to thrive (less than the third percentile at initial presentation). He was started on an airway clearance regimen, pancreatic enzyme supplements, and fat-soluble vitamin supplementation. A routine cough swab at five months of age grew pseudomonas aeruginosa, which was treated with oral ciprofloxacin, and he was nebulized with tobramycin. Repeat cultures after eradication treatment were negative. Routine CF investigations were unremarkable except for mildly elevated aspartate aminotransferase (AST) (99 units/L). Chest X-ray did not show any abnormality, and ultrasound abdomen revealed a mild increase in liver size and prominent periportal tracts (Figure 1).

Based on the clinical presentation and screening test results, a full CFTR gene (NM_000492.3) sequencing was done, which identified a novel frameshift variant, c.3623del, in apparent homozygosity. This variant was previously identified in the homozygous state in only one male patient with cystic fibrosis but was absent from large population studies such as the genome aggregation database (gnomAD) and the Greater Middle East (GME) Variome database. The child was subsequently followed up in our department at frequent intervals. He did not have any recurrent pulmonary exacerbations or pseudomonas regrowth. Pancreatic elastase was persistently low even after adjusting the pancreatic enzyme doses. At present, he is gaining weight (weight at 25th centile), has no recent pulmonary exacerbations, and is on a regular airway clearance regimen.

## Discussion

Cystic fibrosis is a recessively inherited disorder caused by mutations in the CFTR gene on chromosome 7q 31. More than 1900 mutations were identified, of which 1500 are potential causes of cystic fibrosis in patients suspected to have the disease [6]. A delta F508 mutation in the CFTR gene is the most common, which accounts for two-thirds of the total CFTR mutations. The highest incidence of CF worldwide has been observed in European whites and Ashkenazi Jews, at one in 2500 and one in 2270, respectively [7]. Several mutations are rare and may be restricted to certain populations. Their effects on clinical phenotypes are understudied.

The mutation (c.3623del (p.Gly1208Alafsx3)) legacy name 3755 del G identified in our patient is the second case in the CFTR database, and the first case reported in detail. The c.3623del (p.Gly1208Alafsx3) variant detected is in apparent homozygosity in the CFTR gene. This defect has been reported in the Cystic Fibrosis Mutation Database (CFTR1) and Universal Mutation Database (UMD)-CFTR database with one homozygous patient with cystic fibrosis reported [8]. This frameshift variant is predicted to alter the protein's amino acid sequence beginning at position 1208 and lead to a premature termination codon 3 amino acids downstream (p.Gly1208Alafs*s). This alteration, which affects all known biologically relevant CFTR transcripts, is then predicted to lead to a truncated or absent protein. The bi-allelic loss of function of the CFTR gene is a well-established disease mechanism in cystic fibrosis. Therefore, following the American College of Medical Genetics and Genomics sequence variant interpretation guidelines, this variant was classified as pathogenic and is considered the underlying molecular diagnosis for this patient [9]. This variant is apparently homozygous, although a large deletion of the second allele could not be ruled out as parental testing was not conducted.

From the available data, the first identified child is from Lebanon and presented as atypical CF and with pancreatic insufficiency. Our patient presented with classic CF, with pseudomonas infection, pancreatic insufficiency, and abnormal sweat test. He is two years old now and doing well, and it is too early to predict the prognosis of his disease.

It has been proved that there are rare CFTR mutations, which are specific to some ethnic groups [10]. Studies have recommended a diverse mutation screen analysis, including ethnic‐specific mutation testing when screening for mutations in the diverse population. In fact, the 23-mutation panel (American College of Medical Genetics and Genomics) that is used for diagnosis and newborn screening in the general population detects fewer than half of the molecular mutations of patients with CF who are neither Caucasian nor Native Americans [11]. In our case, the genetic testing for 139 mutations done in Germany could not identify any defect. Only the CFTR gene sequencing identified the above pathogenic variant. This reinforces the practice for a broad range of CFTR mutation analyses, which is currently feasible and cost-effective using the next-generation sequencing technology, for some ethnic groups who do not have their mutations identified with the standard mutation panels [12].

Early diagnosis and initiation of treatment are important in the management of CF, which improves the ultimate outcome of the child with the best outcome if the initiation is before two months of age [13]. Therefore, children with a suspicion of CF should undergo full sequencing even if the initial screening for common mutations is negative. Further research to categorize this mutation and understand its effect on CFTR protein dysfunction is needed. It will also be interesting to know whether the CFTR potentiators and modulators would be beneficial in such patients in the future.

## Conclusions

CF is a life-threatening condition where an early diagnosis can make a drastic improvement in the quality of life of the patients. The routine genetic mutations analysis may not identify some of the rare ethnic-specific mutations. Comprehensive CFTR genetic analysis is needed to identify such mutations and to decide on specific targeted therapy.
